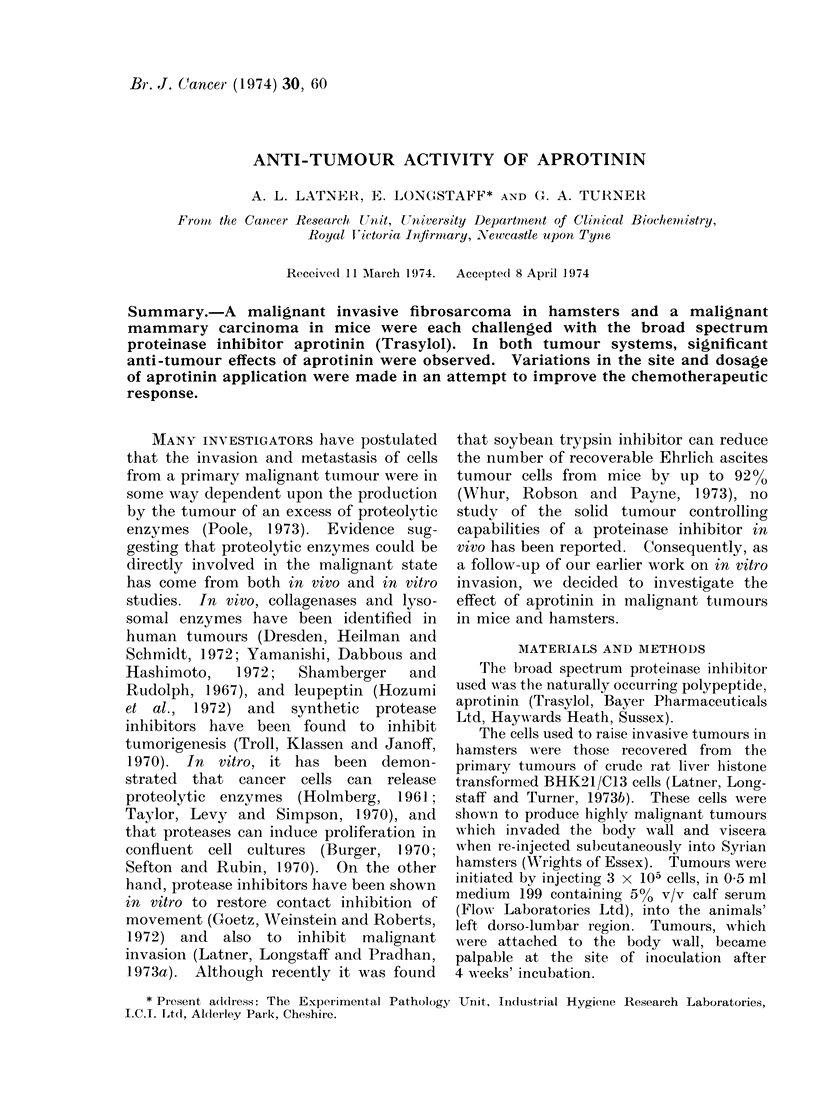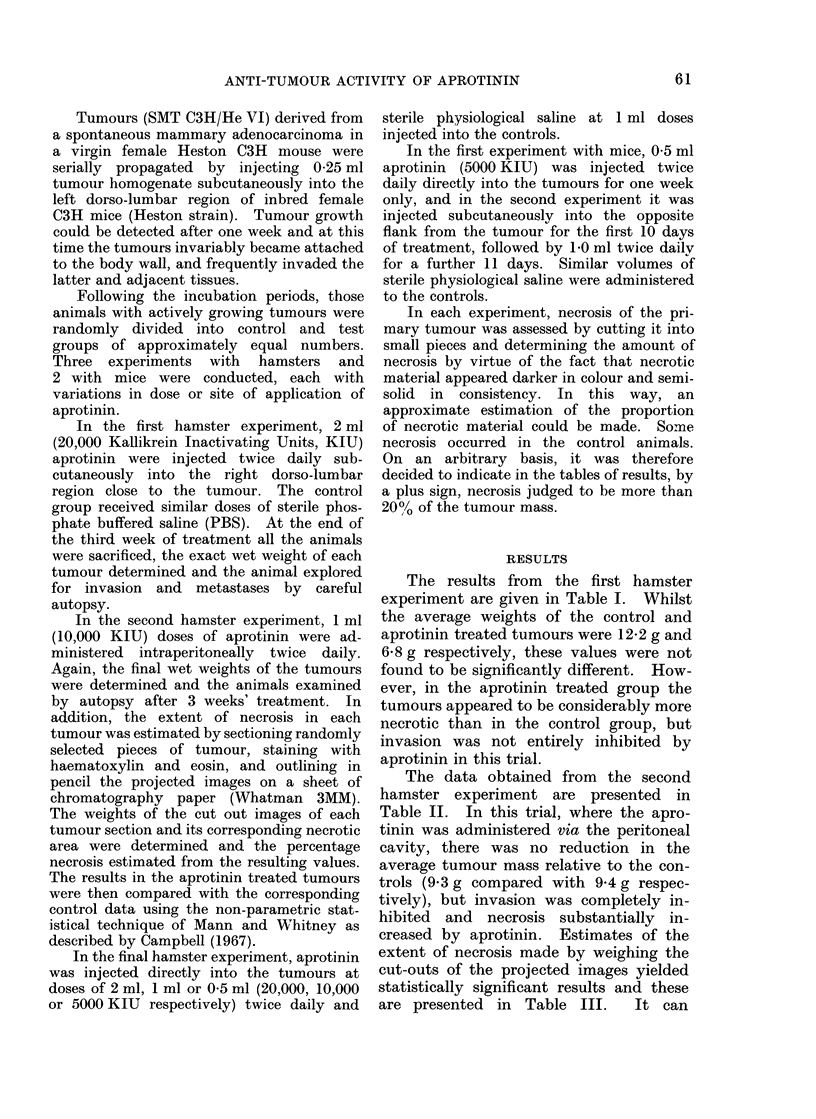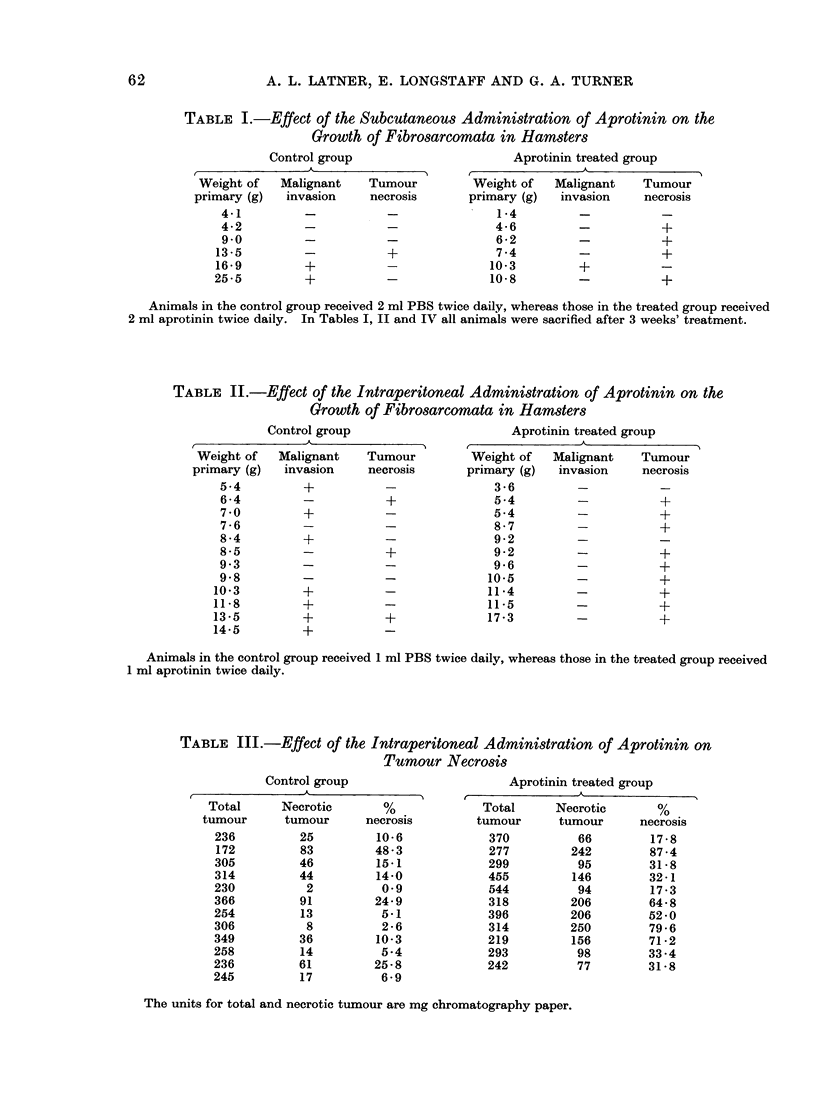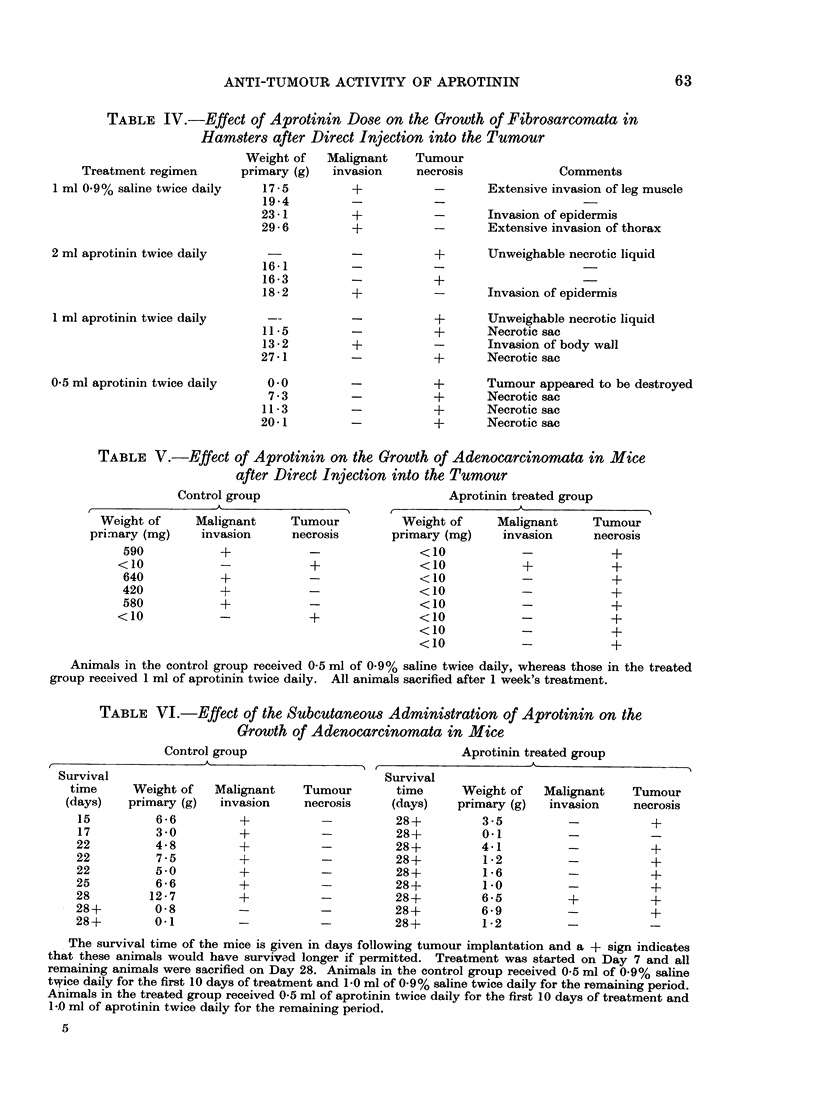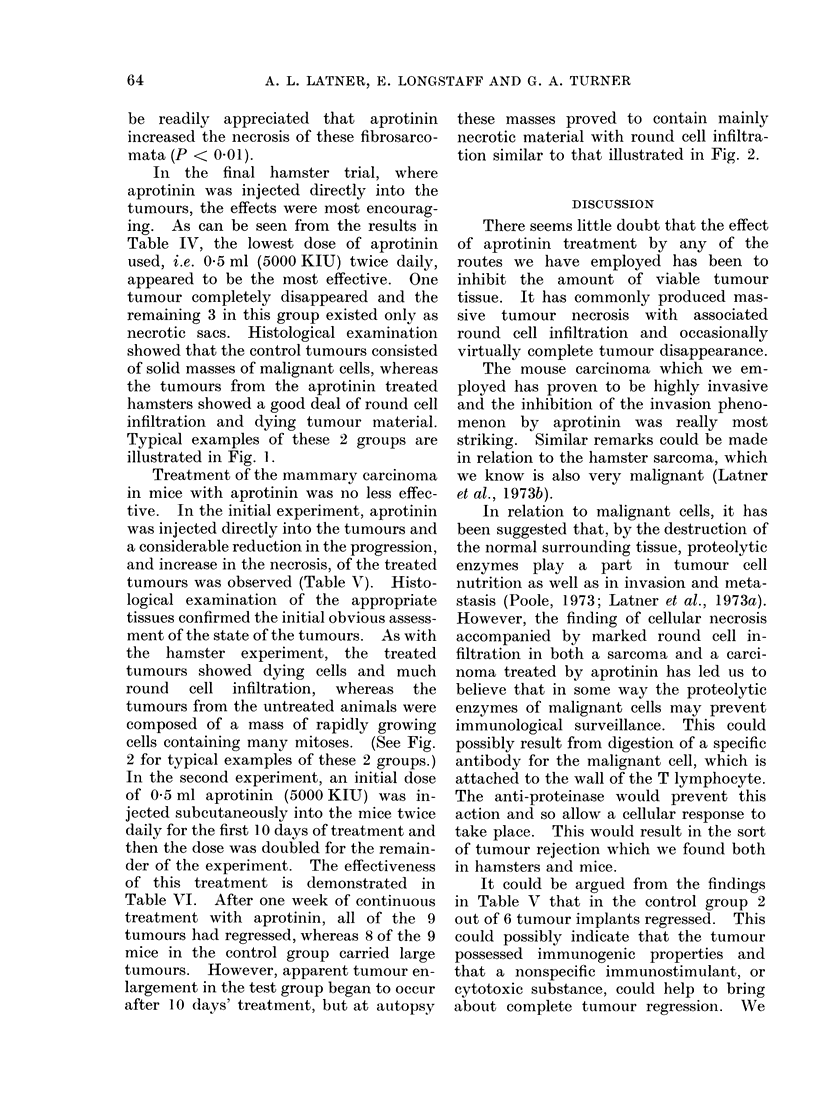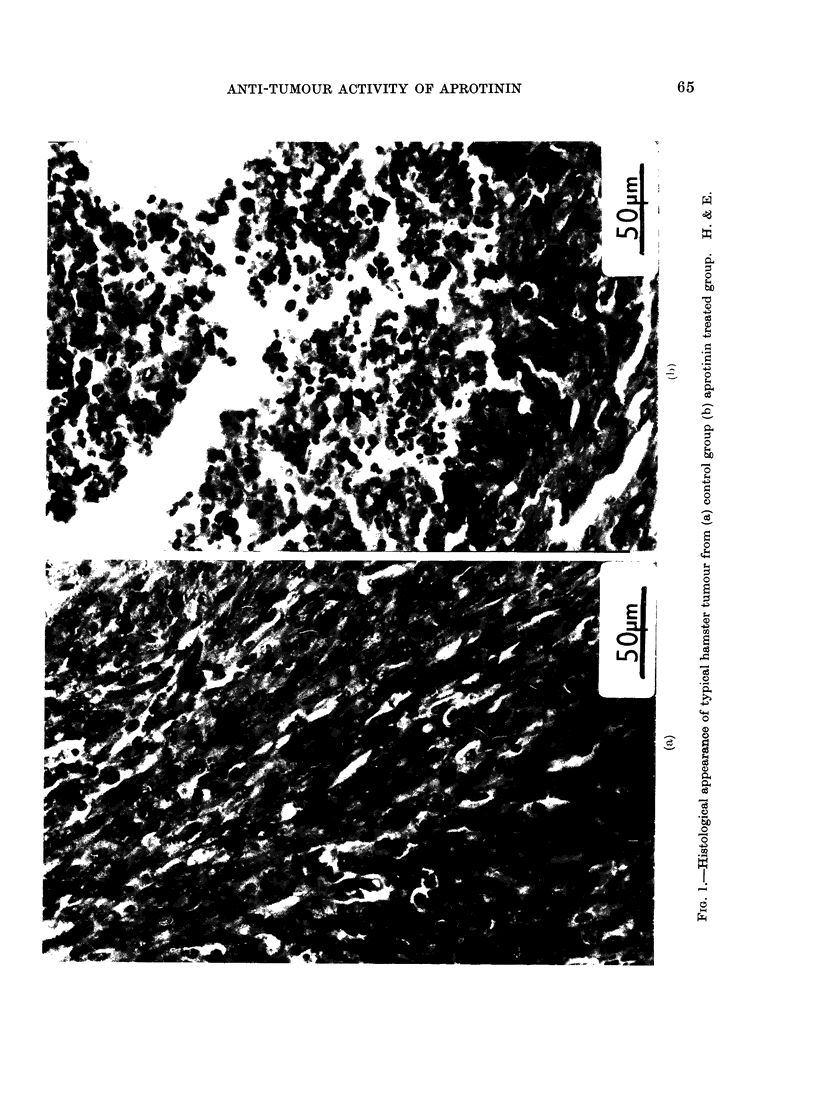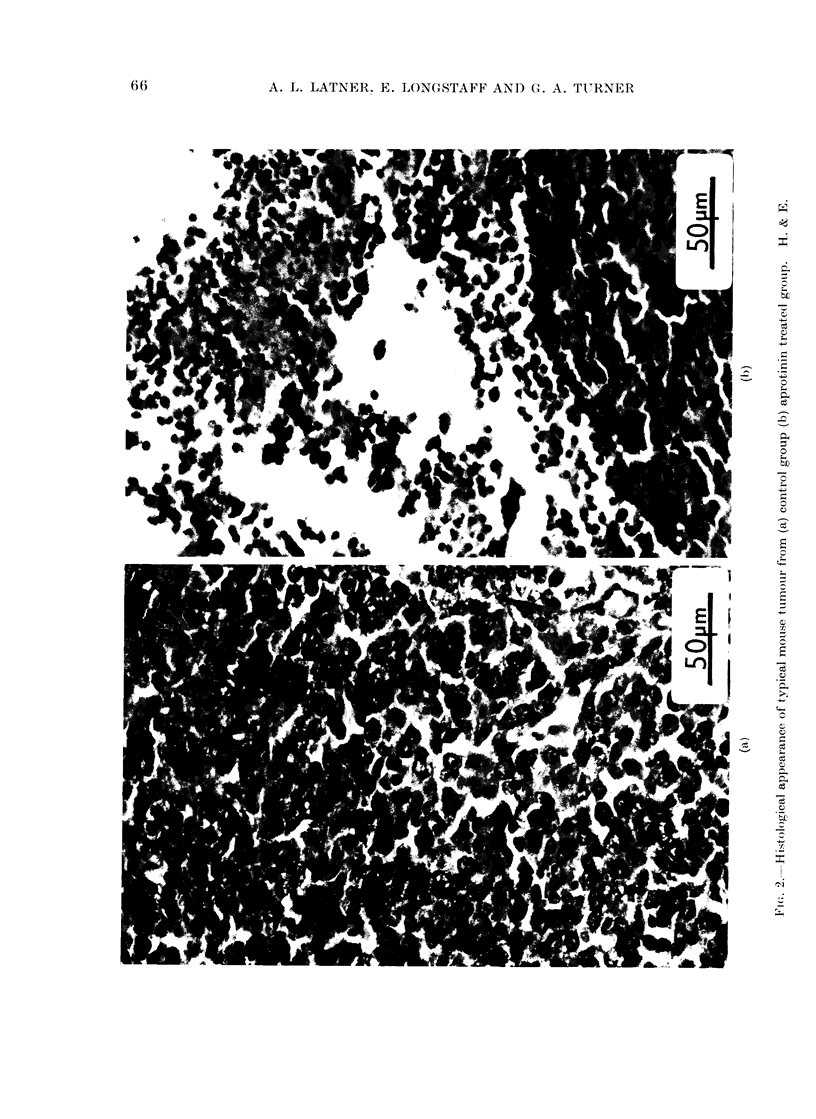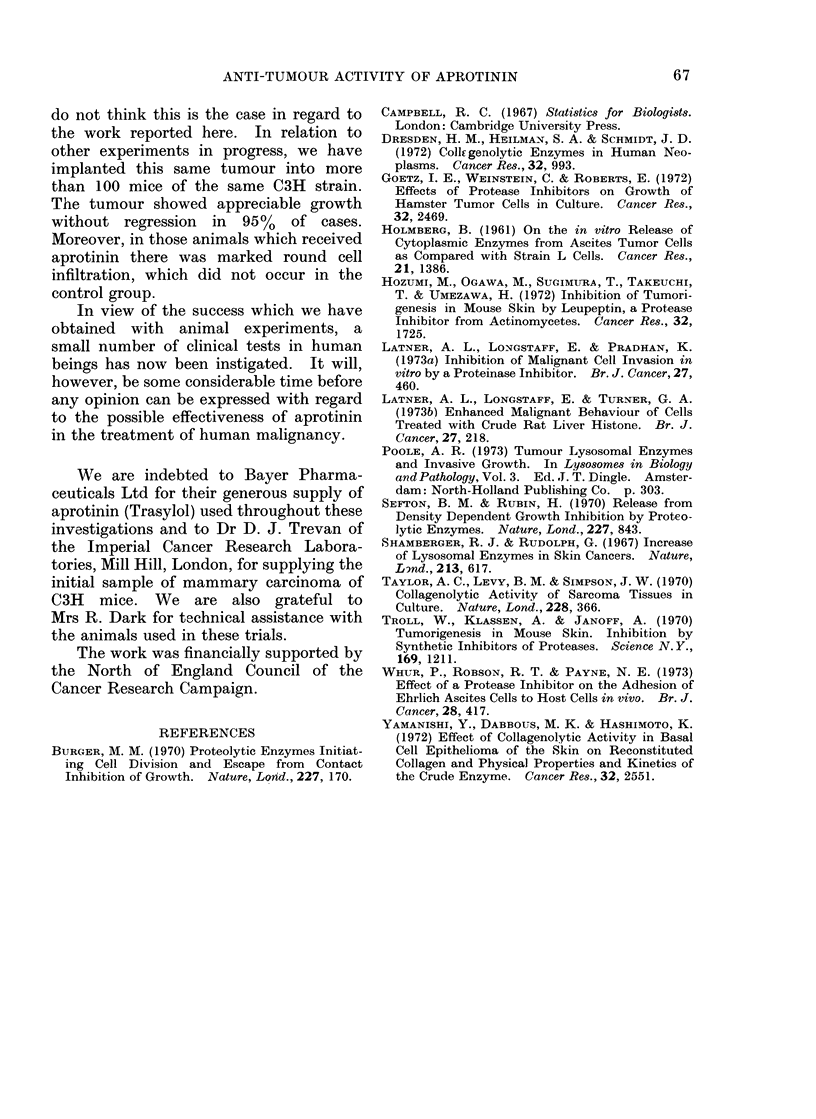# Anti-tumour Activity of Aprotinin

**DOI:** 10.1038/bjc.1974.113

**Published:** 1974-07

**Authors:** A. L. Latner, E. Longstaff, G. A. Turner

## Abstract

**Images:**


*Br. J. Cancer* (1974) **30**, 60

### ANTI-TUMOUR ACTIVITY OF APROTININ

A. L. LATNER, E. LONGSTAFF* AND G. A. TURNER

*From the Cancer Research Unit, University Department of Clinical Biochemistry, Royal Victoria Infirmary, Newcastle upon Tyne*

Received 11 March 1974. Accepted 8 April 1974

**Summary.—A malignant invasive fibrosarcoma in hamsters and a malignant mammary carcinoma in mice were each challenged with the broad spectrum proteinase inhibitor aprotinin (Trasylol). In both tumour systems, significant anti-tumour effects of aprotinin were observed. Variations in the site and dosage of aprotinin application were made in an attempt to improve the chemotherapeutic response.**

MANY INVESTIGATORS have postulated that the invasion and metastasis of cells from a primary malignant tumour were in some way dependent upon the production by the tumour of an excess of proteolytic enzymes (Poole, 1973). Evidence suggesting that proteolytic enzymes could be directly involved in the malignant state has come from both *in vivo* and *in vitro* studies. *In vivo*, collagenases and lysosomal enzymes have been identified in human tumours (Dresden, Heilman and Schmidt, 1972; Yamanishi, Dabbous and Hashimoto, 1972; Shamberger and Rudolph, 1967), and leupeptin (Hozumi *et al.*, 1972) and synthetic protease inhibitors have been found to inhibit tumorigenesis (Troll, Klassen and Janoff, 1970). *In vitro*, it has been demonstrated that cancer cells can release proteolytic enzymes (Holmberg, 1961; Taylor, Levy and Simpson, 1970), and that proteases can induce proliferation in confluent cell cultures (Burger, 1970; Sefton and Rubin, 1970). On the other hand, protease inhibitors have been shown *in vitro* to restore contact inhibition of movement (Goetz, Weinstein and Roberts, 1972) and also to inhibit malignant invasion (Latner, Longstaff and Pradhan, 1973*a*). Although recently it was found that soybean trypsin inhibitor can reduce the number of recoverable Ehrlich ascites tumour cells from mice by up to 92% (Whur, Robson and Payne, 1973), no study of the solid tumour controlling capabilities of a proteinase inhibitor *in vivo* has been reported. Consequently, as a follow-up of our earlier work on *in vitro* invasion, we decided to investigate the effect of aprotinin in malignant tumours in mice and hamsters.

### MATERIALS AND METHODS

The broad spectrum proteinase inhibitor used was the naturally occurring polypeptide, aprotinin (Trasylol, Bayer Pharmaceuticals Ltd, Haywards Heath, Sussex).

The cells used to raise invasive tumours in hamsters were those recovered from the primary tumours of crude rat liver histone transformed BHK21/C13 cells (Latner, Longstaff and Turner, 1973*b*). These cells were shown to produce highly malignant tumours which invaded the body wall and viscera when re-injected subcutaneously into Syrian hamsters (Wrights of Essex). Tumours were initiated by injecting $3 \times 10^5$ cells, in 0·5 ml medium 199 containing 5% v/v calf serum (Flow Laboratories Ltd), into the animals' left dorso-lumbar region. Tumours, which were attached to the body wall, became palpable at the site of inoculation after 4 weeks' incubation.

* Present address: The Experimental Pathology Unit, Industrial Hygiene Research Laboratories, I.C.I. Ltd, Alderley Park, Cheshire.

Tumours (SMT C3H/He VI) derived from a spontaneous mammary adenocarcinoma in a virgin female Heston C3H mouse were serially propagated by injecting 0·25 ml tumour homogenate subcutaneously into the left dorso-lumbar region of inbred female C3H mice (Heston strain). Tumour growth could be detected after one week and at this time the tumours invariably became attached to the body wall, and frequently invaded the latter and adjacent tissues.

Following the incubation periods, those animals with actively growing tumours were randomly divided into control and test groups of approximately equal numbers. Three experiments with hamsters and 2 with mice were conducted, each with variations in dose or site of application of aprotinin.

In the first hamster experiment, 2 ml (20,000 Kallikrein Inactivating Units, KIU) aprotinin were injected twice daily subcutaneously into the right dorso-lumbar region close to the tumour. The control group received similar doses of sterile phosphate buffered saline (PBS). At the end of the third week of treatment all the animals were sacrificed, the exact wet weight of each tumour determined and the animal explored for invasion and metastases by careful autopsy.

In the second hamster experiment, 1 ml (10,000 KIU) doses of aprotinin were administered intraperitoneally twice daily. Again, the final wet weights of the tumours were determined and the animals examined by autopsy after 3 weeks' treatment. In addition, the extent of necrosis in each tumour was estimated by sectioning randomly selected pieces of tumour, staining with haematoxylin and eosin, and outlining in pencil the projected images on a sheet of chromatography paper (Whatman 3MM). The weights of the cut out images of each tumour section and its corresponding necrotic area were determined and the percentage necrosis estimated from the resulting values. The results in the aprotinin treated tumours were then compared with the corresponding control data using the non-parametric statistical technique of Mann and Whitney as described by Campbell (1967).

In the final hamster experiment, aprotinin was injected directly into the tumours at doses of 2 ml, 1 ml or 0·5 ml (20,000, 10,000 or 5000 KIU respectively) twice daily and sterile physiological saline at 1 ml doses injected into the controls.

In the first experiment with mice, 0·5 ml aprotinin (5000 KIU) was injected twice daily directly into the tumours for one week only, and in the second experiment it was injected subcutaneously into the opposite flank from the tumour for the first 10 days of treatment, followed by 1·0 ml twice daily for a further 11 days. Similar volumes of sterile physiological saline were administered to the controls.

In each experiment, necrosis of the primary tumour was assessed by cutting it into small pieces and determining the amount of necrosis by virtue of the fact that necrotic material appeared darker in colour and semisolid in consistency. In this way, an approximate estimation of the proportion of necrotic material could be made. Some necrosis occurred in the control animals. On an arbitrary basis, it was therefore decided to indicate in the tables of results, by a plus sign, necrosis judged to be more than 20% of the tumour mass.

### RESULTS

The results from the first hamster experiment are given in Table I. Whilst the average weights of the control and aprotinin treated tumours were 12·2 g and 6·8 g respectively, these values were not found to be significantly different. However, in the aprotinin treated group the tumours appeared to be considerably more necrotic than in the control group, but invasion was not entirely inhibited by aprotinin in this trial.

The data obtained from the second hamster experiment are presented in Table II. In this trial, where the aprotinin was administered *via* the peritoneal cavity, there was no reduction in the average tumour mass relative to the controls (9·3 g compared with 9·4 g respectively), but invasion was completely inhibited and necrosis substantially increased by aprotinin. Estimates of the extent of necrosis made by weighing the cut-outs of the projected images yielded statistically significant results and these are presented in Table III. It can

TABLE I.—*Effect of the Subcutaneous Administration of Aprotinin on the Growth of Fibrosarcomata in Hamsters*

| Control group | | | Aprotinin treated group | | |
|---|---|---|---|---|---|
| Weight of primary (g) | Malignant invasion | Tumour necrosis | Weight of primary (g) | Malignant invasion | Tumour necrosis |
| 4·1 | − | − | 1·4 | − | − |
| 4·2 | − | − | 4·6 | − | + |
| 9·0 | − | − | 6·2 | − | + |
| 13·5 | − | + | 7·4 | − | + |
| 16·9 | + | − | 10·3 | + | − |
| 25·5 | + | − | 10·8 | − | + |

Animals in the control group received 2 ml PBS twice daily, whereas those in the treated group received 2 ml aprotinin twice daily. In Tables I, II and IV all animals were sacrified after 3 weeks' treatment.

TABLE II.—*Effect of the Intraperitoneal Administration of Aprotinin on the Growth of Fibrosarcomata in Hamsters*

| Control group | | | Aprotinin treated group | | |
|---|---|---|---|---|---|
| Weight of primary (g) | Malignant invasion | Tumour necrosis | Weight of primary (g) | Malignant invasion | Tumour necrosis |
| 5·4 | + | − | 3·6 | − | − |
| 6·4 | − | + | 5·4 | − | + |
| 7·0 | + | − | 5·4 | − | + |
| 7·6 | − | − | 8·7 | − | + |
| 8·4 | + | − | 9·2 | − | − |
| 8·5 | − | + | 9·2 | − | + |
| 9·3 | − | − | 9·6 | − | + |
| 9·8 | − | − | 10·5 | − | + |
| 10·3 | + | − | 11·4 | − | + |
| 11·8 | + | − | 11·5 | − | + |
| 13·5 | + | + | 17·3 | − | + |
| 14·5 | + | − | | | |

Animals in the control group received 1 ml PBS twice daily, whereas those in the treated group received 1 ml aprotinin twice daily.

TABLE III.—*Effect of the Intraperitoneal Administration of Aprotinin on Tumour Necrosis*

| Control group | | | Aprotinin treated group | | |
|---|---|---|---|---|---|
| Total tumour | Necrotic tumour | % necrosis | Total tumour | Necrotic tumour | % necrosis |
| 236 | 25 | 10·6 | 370 | 66 | 17·8 |
| 172 | 83 | 48·3 | 277 | 242 | 87·4 |
| 305 | 46 | 15·1 | 299 | 95 | 31·8 |
| 314 | 44 | 14·0 | 455 | 146 | 32·1 |
| 230 | 2 | 0·9 | 544 | 94 | 17·3 |
| 366 | 91 | 24·9 | 318 | 206 | 64·8 |
| 254 | 13 | 5·1 | 396 | 206 | 52·0 |
| 306 | 8 | 2·6 | 314 | 250 | 79·6 |
| 349 | 36 | 10·3 | 219 | 156 | 71·2 |
| 258 | 14 | 5·4 | 293 | 98 | 33·4 |
| 236 | 61 | 25·8 | 242 | 77 | 31·8 |
| 245 | 17 | 6·9 | | | |

The units for total and necrotic tumour are mg chromatography paper.

TABLE IV.—*Effect of Aprotinin Dose on the Growth of Fibrosarcomata in Hamsters after Direct Injection into the Tumour*

| Treatment regimen | Weight of primary (g) | Malignant invasion | Tumour necrosis | Comments |
|---|---|---|---|---|
| 1 ml 0·9% saline twice daily | 17·5 | + | — | Extensive invasion of leg muscle |
| | 19·4 | — | — | — |
| | 23·1 | + | — | Invasion of epidermis |
| | 29·6 | + | — | Extensive invasion of thorax |
| 2 ml aprotinin twice daily | — | — | + | Unweighable necrotic liquid |
| | 16·1 | — | — | — |
| | 16·3 | — | + | — |
| | 18·2 | + | — | Invasion of epidermis |
| 1 ml aprotinin twice daily | — | — | + | Unweighable necrotic liquid |
| | 11·5 | — | + | Necrotic sac |
| | 13·2 | + | — | Invasion of body wall |
| | 27·1 | — | + | Necrotic sac |
| 0·5 ml aprotinin twice daily | 0·0 | — | + | Tumour appeared to be destroyed |
| | 7·3 | — | + | Necrotic sac |
| | 11·3 | — | + | Necrotic sac |
| | 20·1 | — | + | Necrotic sac |

TABLE V.—*Effect of Aprotinin on the Growth of Adenocarcinomata in Mice after Direct Injection into the Tumour*

| Control group | | | Aprotinin treated group | | |
|---|---|---|---|---|---|
| Weight of primary (mg) | Malignant invasion | Tumour necrosis | Weight of primary (mg) | Malignant invasion | Tumour necrosis |
| 590 | + | — | <10 | — | + |
| <10 | — | + | <10 | + | + |
| 640 | + | — | <10 | — | + |
| 420 | + | — | <10 | — | + |
| 580 | + | — | <10 | — | + |
| <10 | — | + | <10 | — | + |
| | | | <10 | — | + |
| | | | <10 | — | + |

Animals in the control group received 0·5 ml of 0·9% saline twice daily, whereas those in the treated group received 1 ml of aprotinin twice daily. All animals sacrified after 1 week's treatment.

TABLE VI.—*Effect of the Subcutaneous Administration of Aprotinin on the Growth of Adenocarcinomata in Mice*

| Control group | | | | Aprotinin treated group | | | |
|---|---|---|---|---|---|---|---|
| Survival time (days) | Weight of primary (g) | Malignant invasion | Tumour necrosis | Survival time (days) | Weight of primary (g) | Malignant invasion | Tumour necrosis |
| 15 | 6·6 | + | — | 28+ | 3·5 | — | + |
| 17 | 3·0 | + | — | 28+ | 0·1 | — | — |
| 22 | 4·8 | + | — | 28+ | 4·1 | — | + |
| 22 | 7·5 | + | — | 28+ | 1·2 | — | + |
| 22 | 5·0 | + | — | 28+ | 1·6 | — | + |
| 25 | 6·6 | + | — | 28+ | 1·0 | — | + |
| 28 | 12·7 | + | — | 28+ | 6·5 | + | + |
| 28+ | 0·8 | — | — | 28+ | 6·9 | — | + |
| 28+ | 0·1 | — | — | 28+ | 1·2 | — | — |

The survival time of the mice is given in days following tumour implantation and a + sign indicates that these animals would have survived longer if permitted. Treatment was started on Day 7 and all remaining animals were sacrified on Day 28. Animals in the control group received 0·5 ml of 0·9% saline twice daily for the first 10 days of treatment and 1·0 ml of 0·9% saline twice daily for the remaining period. Animals in the treated group received 0·5 ml of aprotinin twice daily for the first 10 days of treatment and 1·0 ml of aprotinin twice daily for the remaining period.

be readily appreciated that aprotinin increased the necrosis of these fibrosarcomata ($P < 0{\cdot}01$).

In the final hamster trial, where aprotinin was injected directly into the tumours, the effects were most encouraging. As can be seen from the results in Table IV, the lowest dose of aprotinin used, *i.e.* 0·5 ml (5000 KIU) twice daily, appeared to be the most effective. One tumour completely disappeared and the remaining 3 in this group existed only as necrotic sacs. Histological examination showed that the control tumours consisted of solid masses of malignant cells, whereas the tumours from the aprotinin treated hamsters showed a good deal of round cell infiltration and dying tumour material. Typical examples of these 2 groups are illustrated in Fig. 1.

Treatment of the mammary carcinoma in mice with aprotinin was no less effective. In the initial experiment, aprotinin was injected directly into the tumours and a considerable reduction in the progression, and increase in the necrosis, of the treated tumours was observed (Table V). Histological examination of the appropriate tissues confirmed the initial obvious assessment of the state of the tumours. As with the hamster experiment, the treated tumours showed dying cells and much round cell infiltration, whereas the tumours from the untreated animals were composed of a mass of rapidly growing cells containing many mitoses. (See Fig. 2 for typical examples of these 2 groups.) In the second experiment, an initial dose of 0·5 ml aprotinin (5000 KIU) was injected subcutaneously into the mice twice daily for the first 10 days of treatment and then the dose was doubled for the remainder of the experiment. The effectiveness of this treatment is demonstrated in Table VI. After one week of continuous treatment with aprotinin, all of the 9 tumours had regressed, whereas 8 of the 9 mice in the control group carried large tumours. However, apparent tumour enlargement in the test group began to occur after 10 days' treatment, but at autopsy these masses proved to contain mainly necrotic material with round cell infiltration similar to that illustrated in Fig. 2.

### DISCUSSION

There seems little doubt that the effect of aprotinin treatment by any of the routes we have employed has been to inhibit the amount of viable tumour tissue. It has commonly produced massive tumour necrosis with associated round cell infiltration and occasionally virtually complete tumour disappearance.

The mouse carcinoma which we employed has proven to be highly invasive and the inhibition of the invasion phenomenon by aprotinin was really most striking. Similar remarks could be made in relation to the hamster sarcoma, which we know is also very malignant (Latner *et al.*, 1973*b*).

In relation to malignant cells, it has been suggested that, by the destruction of the normal surrounding tissue, proteolytic enzymes play a part in tumour cell nutrition as well as in invasion and metastasis (Poole, 1973; Latner *et al.*, 1973*a*). However, the finding of cellular necrosis accompanied by marked round cell infiltration in both a sarcoma and a carcinoma treated by aprotinin has led us to believe that in some way the proteolytic enzymes of malignant cells may prevent immunological surveillance. This could possibly result from digestion of a specific antibody for the malignant cell, which is attached to the wall of the T lymphocyte. The anti-proteinase would prevent this action and so allow a cellular response to take place. This would result in the sort of tumour rejection which we found both in hamsters and mice.

It could be argued from the findings in Table V that in the control group 2 out of 6 tumour implants regressed. This could possibly indicate that the tumour possessed immunogenic properties and that a nonspecific immunostimulant, or cytotoxic substance, could help to bring about complete tumour regression. We

FIG. 1.—Histological appearance of typical hamster tumour from (a) control group (b) aprotinin treated group. H. & E.

FIG. 2.—Histological appearance of typical mouse tumour from (a) control group (b) aprotinin treated group. H. & E.

do not think this is the case in regard to the work reported here. In relation to other experiments in progress, we have implanted this same tumour into more than 100 mice of the same C3H strain. The tumour showed appreciable growth without regression in 95% of cases. Moreover, in those animals which received aprotinin there was marked round cell infiltration, which did not occur in the control group.

In view of the success which we have obtained with animal experiments, a small number of clinical tests in human beings has now been instigated. It will, however, be some considerable time before any opinion can be expressed with regard to the possible effectiveness of aprotinin in the treatment of human malignancy.

We are indebted to Bayer Pharmaceuticals Ltd for their generous supply of aprotinin (Trasylol) used throughout these investigations and to Dr D. J. Trevan of the Imperial Cancer Research Laboratories, Mill Hill, London, for supplying the initial sample of mammary carcinoma of C3H mice. We are also grateful to Mrs R. Dark for technical assistance with the animals used in these trials.

The work was financially supported by the North of England Council of the Cancer Research Campaign.

### REFERENCES

BURGER, M. M. (1970) Proteolytic Enzymes Initiating Cell Division and Escape from Contact Inhibition of Growth. *Nature, Lond.*, **227**, 170.

CAMPBELL, R. C. (1967) *Statistics for Biologists*. London: Cambridge University Press.

DRESDEN, H. M., HEILMAN, S. A. & SCHMIDT, J. D. (1972) Collagenolytic Enzymes in Human Neoplasms. *Cancer Res.*, **32**, 993.

GOETZ, I. E., WEINSTEIN, C. & ROBERTS, E. (1972) Effects of Protease Inhibitors on Growth of Hamster Tumor Cells in Culture. *Cancer Res.*, **32**, 2469.

HOLMBERG, B. (1961) On the *in vitro* Release of Cytoplasmic Enzymes from Ascites Tumor Cells as Compared with Strain L Cells. *Cancer Res.*, **21**, 1386.

HOZUMI, M., OGAWA, M., SUGIMURA, T., TAKEUCHI, T. & UMEZAWA, H. (1972) Inhibition of Tumorigenesis in Mouse Skin by Leupeptin, a Protease Inhibitor from Actinomycetes. *Cancer Res.*, **32**, 1725.

LATNER, A. L., LONGSTAFF, E. & PRADHAN, K. (1973*a*) Inhibition of Malignant Cell Invasion *in vitro* by a Proteinase Inhibitor. *Br. J. Cancer*, **27**, 460.

LATNER, A. L., LONGSTAFF, E. & TURNER, G. A. (1973*b*) Enhanced Malignant Behaviour of Cells Treated with Crude Rat Liver Histone. *Br. J. Cancer*, **27**, 218.

POOLE, A. R. (1973) Tumour Lysosomal Enzymes and Invasive Growth. In *Lysosomes in Biology and Pathology*, Vol. 3. Ed. J. T. Dingle. Amsterdam: North-Holland Publishing Co. p. 303.

SEFTON, B. M. & RUBIN, H. (1970) Release from Density Dependent Growth Inhibition by Proteolytic Enzymes. *Nature, Lond.*, **227**, 843.

SHAMBERGER, R. J. & RUDOLPH, G. (1967) Increase of Lysosomal Enzymes in Skin Cancers. *Nature, Lond.*, **213**, 617.

TAYLOR, A. C., LEVY, B. M. & SIMPSON, J. W. (1970) Collagenolytic Activity of Sarcoma Tissues in Culture. *Nature, Lond.*, **228**, 366.

TROLL, W., KLASSEN, A. & JANOFF, A. (1970) Tumorigenesis in Mouse Skin. Inhibition by Synthetic Inhibitors of Proteases. *Science N.Y.*, **169**, 1211.

WHUR, P., ROBSON, R. T. & PAYNE, N. E. (1973) Effect of a Protease Inhibitor on the Adhesion of Ehrlich Ascites Cells to Host Cells *in vivo*. *Br. J. Cancer*, **28**, 417.

YAMANISHI, Y., DABBOUS, M. K. & HASHIMOTO, K. (1972) Effect of Collagenolytic Activity in Basal Cell Epithelioma of the Skin on Reconstituted Collagen and Physical Properties and Kinetics of the Crude Enzyme. *Cancer Res.*, **32**, 2551.